# Mesenchymal Stem Cell-Macrophage Crosstalk and Maintenance of Inflammatory Microenvironment Homeostasis

**DOI:** 10.3389/fcell.2021.681171

**Published:** 2021-06-25

**Authors:** Di Lu, Yan Xu, Qiuli Liu, Qi Zhang

**Affiliations:** The Biotherapy Center, The Third Affiliated Hospital of Sun Yat-sen University, Guangzhou, China

**Keywords:** mesenchymal stem cells, macrophage, clinic therapy, microenvironment, homeostasis

## Abstract

Macrophages are involved in almost every aspect of biological systems and include development, homeostasis and repair. Mesenchymal stem cells (MSCs) have good clinical application prospects due to their ability to regulate adaptive and innate immune cells, particularly macrophages, and they have been used successfully for many immune disorders, including inflammatory bowel disease (IBD), acute lung injury, and wound healing, which have been reported as macrophage-mediated disorders. In the present review, we focus on the interaction between MSCs and macrophages and summarize their methods of interaction and communication, such as cell-to-cell contact, soluble factor secretion, and organelle transfer. In addition, we discuss the roles of MSC-macrophage crosstalk in the development of disease and maintenance of homeostasis of inflammatory microenvironments. Finally, we provide optimal strategies for applications in immune-related disease treatments.

## Introduction

Mesenchymal stem cells (MSCs) are observed in a variety of tissues, such as bone marrow, umbilical cord tissue, adipose tissue, and placental tissue (Pittenger et al., 1999), and they have a fibroblast-like morphology and multidirectional differentiation potential and can differentiate into cartilage, osteoblasts, and adipocytes (da Silva Meirelles et al., 2006). More importantly, MSCs can regulate the immune response and anti-inflammatory effects in a specific microenvironment by a number of pathways (da Silva Meirelles et al., 2009). Previous reports showed that MSCs regulate the immune response by interacting with various immune cells (Aggarwal et al., 2014; Caires et al., 2018) and the maturation, differentiation, proliferation and functional activation of peripheral blood mononuclear cells (PBMCs) by secreting regulatory molecules and cytokines (Di Nicola et al., 2002; da Silva Meirelles et al., 2006; Gazdic et al., 2015; Vizoso et al., 2017). Additionally, previous studies have shown that MSCs can regulate adaptive immune cells, such as modulating the proliferation and differentiation of B cells, thereby inhibiting T cell proliferation and cytokine secretion and inducing regulatory T cells (Wang R.X. et al., 2014). Moreover, MSCs also possess the potential to control the immune response from innate immune cells, including monocytes, macrophages, dendritic cells (DCs), natural killer cells (NK cells), etc. (Li et al., 2010; Chiesa et al., 2011; Cahill et al., 2015).

Macrophages are specialized phagocytic cells of the innate immune system that have several diverse functions in homeostatic and immune responses. As scavengers, macrophages constantly move around to remove dead cells, pathogenic microbes and other foreign bodies. As key modulator and effector cells in the immune response, the activation of macrophages influences and responds to the immune system. The concepts of classic and alternative activation, also known as M1 and M2, respectively, have become increasingly widespread (Gordon, 2003; Aggarwal et al., 2014), and growing evidence indicates that macrophages are pivotal for the maintenance of the tissue microenvironment and repair. For example, intestinal macrophages have roles in maintaining tissue homeostasis, particularly restoring tissue homeostasis after inflammation. Liposomes, lipoprotein A4 (LXA4) and specialized pre-degradation mediators (SPM) have been shown to direct macrophage differentiation to alternatively activated macrophages with pro-resolving capacity (Na et al., 2019). These macrophages not only inhibit Th1 and Th17 responses (Maizels, 2005) but also play an important role in re-establishing the epithelial barrier upon disruption of the intestinal epithelium (Fox et al., 2010). Similarly, alveolar macrophages are critical for tissue homeostasis, their polarization is driven by injury, and they regulate lung inflammation and resolution (Jackson et al., 2016; Hu and Christman, 2019). Moreover, macrophages constantly monitor the skin microenvironment and maintain skin homeostasis via their plasticity, such as differentiating into pro-inflammatory macrophages, pro-wound healing macrophages, or pro-resolving macrophages to promote or suppress inflammation and modulate wound healing (Barrientos et al., 2008; Mosser and Edwards, 2008; Yanez et al., 2017; Krzyszczyk et al., 2018). In recent years, increasing attention has been given to the study of MSC-macrophage interactions in tissue homeostasis and damage repair. This review will focus on the regulation of MSCs on macrophages, the counteraction of macrophages on MSCs, and the role of MSC-macrophage crosstalk in the inflammatory microenvironment. The importance of the interaction between the two is the maintenance of organization stability.

## Biology of Macrophages

**FIGURE 1:**
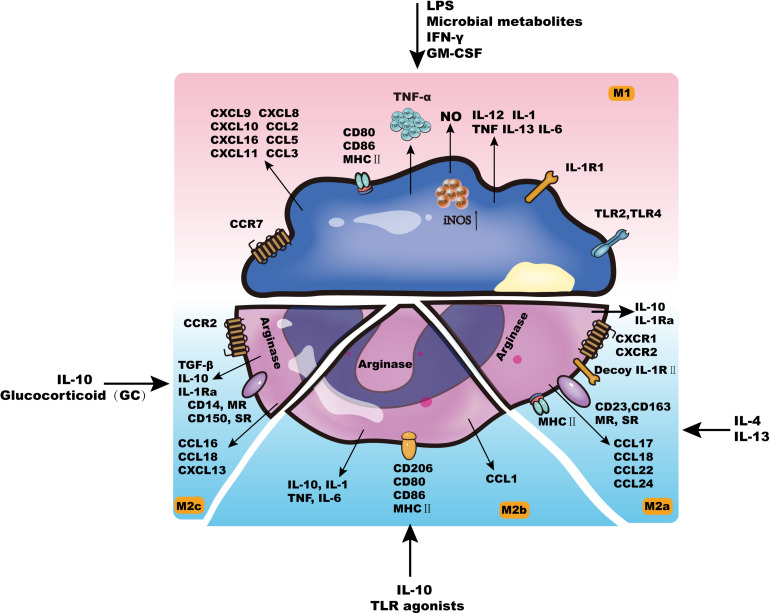
The characteristics of macrophages.

Generally, tissue-resident macrophages are either derived from circulating monocytes or established before birth and maintained long-term (Franken et al., 2016). As early as 1939, Ebert and Florey discovered that monocytes *in vivo* can migrate from the blood to different tissues and organs and then develop and differentiate into macrophages (Mφs) (Willis et al., 2018). The life span of Mφs in different tissues is prolonged, with some able to survive for months to years (Benoit et al., 2008a). Although Mφs have the potential for proliferation in tissues, they rarely divide and are mainly replenished by the continuous migration of monocytes in the blood. According to different activation methods, Mφ can be divided into two categories: classically activated Mφs (M1) and alternatively activated Mφs (M2). These two types have different surface receptor expression, cytokine and chemokine production, effector functions, etc. (Figure 1; Selleri et al., 2016; Heo et al., 2019).

Cytokines such as interferon gamma (IFN-γ), lipopolysaccharide (LPS), granulocyte-macrophage colony stimulating factor (GM-CSF) or tumor necrosis factor (TNF) can activate M1-type cells, which leads to increased self-antigen presentation ability, increased complement-mediated phagocytic activity, proinflammatory factor release (IL-1β, TNF-α, IL-12, IL-6, IL-23, NO, etc.), and chemokine production (CXCL9, CXCL10, etc.) (Benoit et al., 2008b; Biswas et al., 2019). By releasing these inflammatory mediators, M1 cells can promote the elimination of non-self components *in vivo* and play an important role in preventing tumors (Belgiovine et al., 2016). In addition, M1 cells participate in the Th1-mediated immune response as effector cells, thereby promoting inflammation and killing intracellular pathogens (Bystrom et al., 2008; Caires et al., 2016, 2018).

M2-type cells are further divided into M2a, M2b, and M2c cells (Mantovani et al., 2004). M2a can be induced by IL-13 or IL-4, and M2b can be induced by some Toll-like receptor (TLR) ligands and IL-10. M2a and M2b cells mainly perform immune regulation functions by promoting the Th2-mediated immune response (Ben-Mordechai et al., 2013; Ben-Lulu et al., 2014; Behura et al., 2019). The main function of M2c cells is to suppress the occurrence of the immune response, which plays an important role in the process of tissue remodeling (Mantovani et al., 2004).

Complementarily, the recognition and elimination of invading pathogens by macrophages is an important event in the innate immune response. Mφs express a variety of pattern recognition receptors (PRRs), including mannose receptors (MRs), scavenger receptors (SRs), TLRs and phosphatidylserine receptors (PSRs) (Kurokawa et al., 2009). Ligands are highly conserved molecular structures shared by a class or group of specific pathogenic microorganisms and their products, including LPS and lipoteichoic acid (LTA), mannose, peptidoglycan (PGN), bacterial DNA, double-stranded RNA and dextran, which are called pathogen-associated molecular patterns (PAMPs) (Li et al., 2016). When the corresponding PRR and PAMP are combined, a rapid immunobiological response can be mediated. In addition, after Mφ recognizes pathogenic microorganisms via PRR, it is activated at the same time and further produces and secretes a variety of chemokines, cytokines and chemical mediators, including MIP-1α/β, MCP-1, and IL-8, which mediate and promote inflammation. Moreover, Mφs can also secrete a large number of inflammatory mediators, such as leukotrienes, prostaglandins, elastase, lysozyme, and urokinase, to induce and strengthen local inflammation (Peng et al., 2016; Nenasheva et al., 2017). In short, macrophages play an important role in processing and presenting antigens, killing and eliminating intracellular pathogens, participating in antitumor immunity, and even maintaining tissues in environmental homeostasis (Xu et al., 2017; Willis et al., 2018).

## MSCs Modulate the Macrophages

### MSC-Mediated Effects on Macrophages

Previous studies have shown that MSCs have the ability to regulate T cells, B cells, and DC cells (English et al., 2008; Ghannam et al., 2010; Luz-Crawford et al., 2013; Lee et al., 2014). At the same time, the interaction between MSCs and innate immune cells is becoming clearer. MSCs can regulate the polarization, phagocytosis and metabolism of macrophages. Biao Huang’s team found that *in vitro*, MSCs promoted the apoptosis of RAW264.7 cells, a macrophage cell line. They also reported that MSCs can downregulate the number of macrophages in the spleen (Huang et al., 2016). MSCs promote the phagocytosis of macrophages through mitochondrial transfer in distinct diseases, such as acute respiratory distress syndrome (ARDS) and sepsis (Jackson et al., 2016; Jackson and Krasnodembskaya, 2017), although fat-derived MSCs can also reduce the phagocytic capacity of macrophages (Adutler-Lieber et al., 2013). In addition, MSCs promote the differentiation of naive macrophages and enhance their bactericidal effect. These stem cells manipulate the plasticity of macrophages by changing the metabolic state of macrophages through prostaglandin E2 (PGE2) (Vasandan et al., 2016). Studies have shown that MSCs are likely to induce macrophages to transform into an anti-inflammatory/immunosuppressive phenotype (Luz-Crawford et al., 2017). Nakajima and HongLong Zhou reported that MSCs can effectively improve spinal cord injury by polarizing macrophages from the M1 to M2 type (Nakajima et al., 2012; Zhou et al., 2016). Other studies have also reported that MSCs can alleviate liver diseases, such as acute liver injury and liver fibrosis, by inhibiting proinflammatory M1 cells and inducing M2 anti-inflammatory cells (Li C. et al., 2019; Wang et al., 2021). Reports also show that MSCs can improve diabetes by inducing macrophage polarization (Yin et al., 2018; Gao et al., 2019). Liao et al. (2020) demonstrated that MSCs can induce macrophages toward an anti-inflammatory M2 phenotype in ischemic myocardium, and other investigations have illustrated that MSCs can polarize macrophages via direct cell–cell contact or indirectly, such as through soluble factors.

### Mechanism of MSC Regulate the Macrophages

#### Cell–Cell Contact

Mesenchymal stem cells have been reported to affect immune cells through direct cell-to-cell contact (Augello et al., 2005; Gu et al., 2013). Although most reports indicate that the immunomodulatory properties of MSCs depend largely on the secretion of soluble factors, cell-to-cell contact is also an important functional mechanism. Li Y. et al. (2019) found that the direct interaction between M1 macrophages and MSCs is essential for the treatment of LPS-induced abortion by MSCs. Using an abortion model, they demonstrated that MSCs induce immune tolerance based on a TSG-6-dependent paracrine effect and by cell-to-cell contact between MSCs and proinflammatory macrophages (Li Y. et al., 2019). Espagnolle reported that the inhibitory effect of MSCs on T cell proliferation was increased due to the upregulation of CD54 in hMSCs in contact with M1 macrophages (Espagnolle et al., 2017).

#### Soluble Factors

Mesenchymal stem cells can polarize monocytes or M1 macrophages into M2-type macrophages by secreting soluble factors. Previous studies have demonstrated that paracrine activity plays an important role in the anti-inflammatory effect of MSCs (da Silva Meirelles et al., 2009). We have summarized the main soluble factors by which MSCs regulate macrophage polarization.

##### PGE2

Prostaglandin E2 (PGE2) is the main product of arachidonic acid metabolism in mammalian cells and plays an important role in immunosuppression and alleviating inflammation (Peebles, 2019). The Zongjin Li group found that human placenta-derived MSCs can improve 2,4,6-trinitrobenzene sulfonic acid (TNBS)-induced mouse colitis. This therapeutic potency was possibly mediated by MSC production of PGE2 to polarize M2 macrophages (Cao et al., 2020). Wang et al. (2021) reported that MSCs can protect against D-galactosamine (D-Gal)-induced liver failure, and they found that MSCs regulate macrophage polarization by secreting PGE2. In addition, studies have reported that MSCs improve cardiac injury by increasing M2 macrophages through the COX-2-PGE2 pathway (Jin et al., 2019, 2020). In summary, PGE2 is an important factor that mediates MSC polarization of macrophages.

##### TGF-β

Transforming growth factor β (TGF-β) plays a very important role in the immunosuppressive function of MSCs (Eggenhofer et al., 2014). TGF-β, a well-known immunosuppressive factor, can inhibit excessive inflammation (Robertson and Rifkin, 2013; Noh et al., 2016; de Araujo Farias et al., 2018). In addition, TGF-β induces M2 polarization of macrophages and reduces the inflammatory response mediated by macrophages (Li et al., 2006; Byrne et al., 2008; Robertson and Rifkin, 2013). Moreover, Pender et al. (1996) showed that TGF-β improves the survival rate of rats exposed to endotoxic shock by regulating the production of inflammatory mediators in peritoneal macrophages. Liu F. et al. (2019) showed that in a coculture system, TGF-β secreted by MSCs reduced the level of M1 markers, upregulated M2 macrophage marker levels, and inhibited excessive activation of the inflammatory response. Another group reported that TGF-β-licensed MSCs can improve the secretome of inflammatory bone marrow-derived macrophages (Lynch et al., 2020). Based on these findings, TGF-β secreted by MSCs can promote the M2-like polarization of macrophages and improve inflammatory conditions.

##### Indoleamine 2,3-dioxygenase

Indoleamine 2,3-dioxygenase (IDO), an intracellular enzyme, can be induced by interferon-γ (IFN-γ), and it catalyzes tryptophan into kynurenine (Ball et al., 2009). Metabolites of kynurenine have been shown to inhibit T cell proliferation and induce T cell apoptosis (Munn et al., 2005; Fallarino et al., 2006), and it has also been shown to regulate macrophage phenotype. Francois et al. (2012) and other investigators found that MSCs induce the polarization of monocytes toward M2 macropage and reduce monocytes infiltration dependent on IDO activity (Kang J.Y. et al., 2020; Galipeau, 2021; Lim et al., 2021). Lee et al. (2017) also found that melatonin can promote MSCs and improve blood flow perfusion and vessel regeneration by reducing the infiltration of macrophages in an IDO-dependent manner.

##### TSG-6

Tumor necrosis factor-α-induced gene/protein 6 (TSG6) has been reported to reduce inflammatory responses in corneal injury, lung injury, peritonitis and skin wounds (Danchuk et al., 2011; Qi et al., 2014; He et al., 2016). Studies on the effect of MSC-induced TSG-6 expression on macrophages have also been performed. Ko et al. (2016) reported that TSG-6 is required for MSCs to induce a suppressive monocyte/macrophage population. In a mouse colitis model, BM-MSCs had a therapeutic function induced by dextran sodium sulfate (DSS) depending on the production of TSG-6, which polarized macrophages from the M1 to M2 type (Sala et al., 2015). MSCs have also been shown to regulate microglia by secreting TSG-6. In this case, murine BM-MSCs inhibited the production of TNF-a, IL-1β, iNOS, and IL-6 by BV2 microglia stimulated by LPS in a TSG-6-dependent manner (Liu et al., 2014). In short, TSG-6 may play an important role in MSC function related to the programming of macrophages.

##### CCL2 and CXCL12

CCL2 is a chemokine classically associated with the recruitment of macrophages and monocytes during angiogenesis (Dipietro et al., 2001; Khan et al., 2013). MSCs secrete CCL2 at very low concentrations in the resting state (Kinnaird et al., 2004; Zhang et al., 2010). When stimulated with inflammatory cytokines, such as TNFα, MSCs secrete up to 10-fold more CCL2 (Ren et al., 2012). Using a lymphoma transplantation model, Ren et al. (2012) demonstrated that tumor-resident MSCs promote tumor growth by recruiting monocytes/macrophages through CCL2 production. C-X-C motif chemokine ligand 12 (CXCL12) plays a major role in macrophages located in regulatory niches (Sugiyama et al., 2006). Perivascular mesenchymal stem/stromal cells, endothelial cells, mature osteoblasts, and osteoprogenitors can also express CXCL12 (Asada et al., 2017). Papa et al. (2018) demonstrated that MSCs alleviate spinal cord injury through the recruitment of macrophages and conversion of M1 cells to an M2 neuroprotective phenotype by secreting CCL2. Recently, Giri et al. (2020) identified that MSCs can upregulate IL-10 expression in CCR2+ macrophages by secreting CCL2 and CXCL12, which cooperate as a heterodimer *in vitro*.

#### Extracellular Vesicles

An increasing number of studies have shown that MSCs perform many paracrine functions by releasing extracellular vesicles (EVs). Several recently published studies indicated that MSC-based alleviation of colitis mainly relies on MSC-EV-induced suppression of colon macrophages (Wu et al., 2014; Phinney et al., 2015; Cao et al., 2019). Exosomes upregulate the nuclear factor kappaB (NF-κB) signal of macrophages, and the uptake of mitochondria increases their phagocytic capacity (Wu et al., 2014). Cao et al. (2019) showed that MSC-EVs can significantly reduce colitis caused by DSS in mice by inducing colonic macrophage polarization in the immunosuppressive M2 phenotype, and they observed more IL-10-producing M2 macrophages in MSC-EV-treated mice, which was associated with weight loss, intestinal epithelial cell damage and increased colon length. Yang et al. (2015) suggested that MSC-EV effects on the phenotype and function of macrophages occur by the regulating the damaged intestinal antioxidant/oxidant balance. That is, MSC-EV-mediated inhibition of intestinal NO-driven injury is accompanied by a decrease in the activities of myeloperoxidase and malondialdehyde and an increase in the activities of superoxide dismutase and glutathione.

#### Mitochondrial Transfer

Studies have also shown that other mechanisms may have a significant impact on the interaction between MSCs and macrophages. Phinney et al. (2015) described a peculiar process in which macrophages engulf the mitochondria that pass through mitochondrial MSCs and shuttle to the cell membrane. Jackson et al. (2016) also reported that in ARDS, the transfer of MSC mitochondria to macrophages in the lung occurs by tunneling through a nanotube-like structure and direct coculture of MSCs and monocyte-derived macrophages can enhance their swallowing ability (Jackson and Krasnodembskaya, 2017). Morrison et al. (2017) reported that the transfer of mitochondria via MSC-derived EVs promotes phagocytosis and suppresses proinflammatory cytokine secretion by human macrophages. It is worth noting that the above studies have shown that with the uptake of MSC mitochondria, the bioenergy and phagocytic capacity of macrophages are enhanced, which may help increase the clearance of microorganisms in diseases such as pneumonia and sepsis.

## Effect of Macrophage on MSC

**FIGURE 2:**
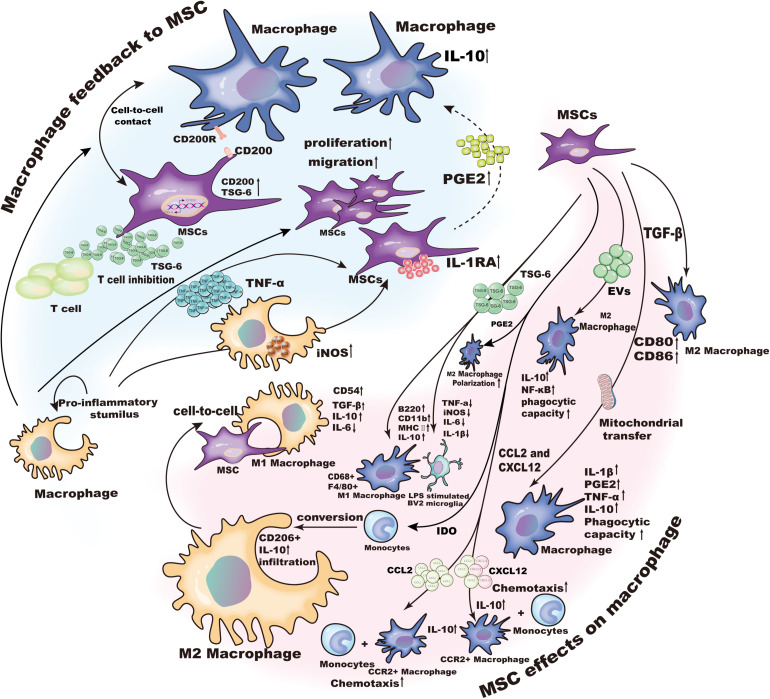
The crosstalk between MSCs and macrophages.

As mentioned above, MSCs can induce the transformation of macrophages. In addition, macrophages, as the target to be regulated, also have a feedback effect on MSCs that includes differentiation, migration, apoptosis and immunomodulatory functions (Figure 2).

### MSC Differentiation

Guihard et al. (2012) found that conditioned media from human monocytes stimulated with LPS or TLR ligands enhanced bone formation by human bone marrow MSCs. Enhanced osteogenesis was also observed by several groups, they demonstrated that M1 macrophages promote osteogenesis in MSCs via the COX-2-PGE2 pathway (Guihard et al., 2012; Lu et al., 2017; Zhang et al., 2017; Nathan et al., 2019; Tang et al., 2019). Recently, the Shi group reported that macrophage-derived supernatants can inhibit the adipogenic differentiation of human adipose-derived mesenchymal stem cells (hADSCs) *in vitro*, irrespective of the polarization status (M0, M1, or M2 macrophages) (Ma et al., 2020). In summary, macrophages also have an important influence on the differentiation ability of MSCs.

### MSC Proliferation, Migration, and Apoptosis

Studies have also clarified that macrophages affect the proliferation, migration, and survival of MSCs, with M2-type macrophages promoting MSC proliferation and migration and M1-type macrophages inducing MSC apoptosis (Yu et al., 2016; Xia Y. et al., 2020). Other reports also demonstrated that M2 macrophages can promote the growth of hMSCs, while M1 type inhibits the growth of hMSCs *in vitro*. In addition, macrophages can mediate the protective and repair effects of MSCs on infarction (Ben-Mordechai et al., 2013; Freytes et al., 2013; Maldonado-Lasuncion et al., 2021). These studies have shown that macrophages can affect MSCs, especially M2 type, and can promote MSCs proliferation and engraftment. This provides new support for targeted macrophage therapy in tissue repair.

### MSC Immunoregulation

In terms of immune regulation, the Nicolas team reported in detail that MSCs express more immune regulatory genes after coculture with macrophages (Lynch et al., 2020). The contact of MSCs with macrophages induces CD54 expression on MSCs and mediates calcium influx in MSCs, thereby promoting the immune regulation function of MSCs (Mercier et al., 1993). de Witte et al. (2018) demonstrated that the phagocytosis of MSCs by monocytes plays a crucial role in the immune regulation of MSCs. Moreover, Li Y. et al. (2019) found that the contact of MSCs with proinflammatory macrophages increases the production of TSG-6 by MSCs, thereby enhancing the inhibitory regulation of MSCs on T cells and macrophages. In addition, proinflammatory macrophages in contact with MSCs upregulated the expression of CD200 on MSCs and promoted the anti-inflammatory transformation of macrophages through the interaction of CD200 and CD200R. The TSG-6-mediated paracrine effect was enhanced by the cell-to-cell contact between MSCs and proinflammatory macrophages (Li Y. et al., 2019).

It is worth noting that macrophage depletion prevents the beneficial effects of MSC-Exos. Similarly, inducing macrophages to produce IL-10 is also partly involved in the beneficial effects of MSC-Exos (Liu H. et al., 2019). Krisztián Németh’s team stated that when mouse-derived bone marrow MSCs are cocultured with macrophages, MSCs will stimulate the release of IL-10 by macrophages through a PGE2-dependent pathway after LPS stimulation. However, in coculture systems, TNF-α and iNOS secreted by macrophages are necessary for MSCs to secrete PGE2 (Nemeth et al., 2009). Reading et al. (2015) suggested that proinflammatory cytokines produced by macrophages stimulate MSCs to produce prostaglandin E2 (PGE2) and interleukin (IL)-1RA, among other immune modulators.

The studies summarized above suggest that after being activated by proinflammatory factors, macrophages secrete cytokines to activate MSCs. After activation, MSCs regulate the immune response and react on macrophages. In the microenvironment of disease, a closed loop interaction occurs between macrophages and MSCs. In other words, MSCs and macrophages present a coordinated relationship to maintain the homeostasis of the inflammatory microenvironment.

## MSC-Macrophage Crosstalk and Microenvironment Homeostasis in Diseases

Macrophages are pivotal for the maintenance of the tissue microenvironment and tissue repair (Ehninger and Trumpp, 2011; Liu et al., 2016). At present, the immunosuppressive function of MSCs derived from bone marrow and cord blood makes them useful in clinical trials and as a treatment method for diseases, including GVHD (Le Blanc and Davies, 2015; Granot et al., 2020), Crohn’s disease (Bamias and Cominelli, 2006), stroke, systemic lupus erythematosus and arthritis (Choi et al., 2012; Steiman et al., 2015) and other diseases. MSCs can improve different diseases by educating macrophages to maintain microenvironment homeostasis (Table 1).

**TABLE 1:** MSC-macrophage crosstalk in different diseases/*in vitro* system.

**Tissue**	**Models**	**MSC-macrophage crosstalk**	**Microenvironment homeostasis**	**References**
Liver	Liver fibrosis	MSC-sEVs induce CX3CR1 + anti-inflammation macrophage	Ameliorated inflammation and fibrosis	Ohara et al., 2018; Kojima et al., 2019; Luo et al., 2019; Song et al., 2020
	Ischemia/Reperfusion (IR)-induced sterile inflammatory liver injury	MSC reprogram macrophage toward anti-inflammatory M2 phenotype	Reduced hepatocellular damage; Diminished liver inflammation	Li C. et al., 2019; Sheng et al., 2021
	Acute liver failure	MSC induced anti-inflammatory (M2) macrophages; reduced levels of macrophage	Ameliorated hepatocyte death and liver inflammatory response.	Li et al., 2017, 2021; Liu et al., 2018; Hwang et al., 2019; Liang et al., 2019; Shao et al., 2020; Wang et al., 2021
	Liver regeneration	Decreased CD68+ macrophages.	Stimulated liver regeneration in rat.	Elchaninov et al., 2018
Heart	Acute rejection of heart transplantation	Inhibited M1 and promoted M2 polarization	Inhibit STAT1 and NF-kB pathways; Inhibit the acute rejection of heart transplantation in mice.	Gao et al., 2020
	Diabetic cardiomyopathy (DCM)	COX-2-PGE2 pathway to promote M2 macrophage polarization	Ameliorate myocardial injury caused by diabetic cardiomyopathy.	Jin et al., 2019, 2020
	Myocardial infarction	Secreting periostin to promote the polarization of M2 macrophage. Reduced pan-macrophage infiltration	Improved cardiac function, decreased infarct size.	Ben-Mordechai et al., 2013; Xu et al., 2019; Gao et al., 2020; Liao et al., 2020
	Atherosclerosis	Stimulate the production of anti-inflammatory factor IL-10, and reduce the production of TNF-α by macrophage.	Reduce atherosclerotic plaque.	Li et al., 2015; Liao et al., 2020
Intestine	Colitis	Chemokine interactome dictates the induction of IL-10+ macrophages and promote M2 polarization.	Mitigate gut injury	Song J.Y. et al., 2017; Song W.J. et al., 2017; Song et al., 2018; Kawata et al., 2019; Liu H. et al., 2019; Cao et al., 2020; Giri et al., 2020
	Inflammatory bowel disease	Up-regulate the expression of IL-10 and promote M2 polarization.	Alleviate inflammatory bowel disease	Wang C. et al., 2014; Mao et al., 2017; Song et al., 2018; Li Y. et al., 2019
Lung	Acute lung injury	Promote M2 macrophage polarization.	Ameliorate acute lung injury induced by LPS.	de Mendonca et al., 2017; Chen et al., 2018; Lv et al., 2020
	Pulmonary arterial	Attenuate the CD68+ macrophage and induce the CD163+ macrophage	Reduce lung inflammation and vascular remodeling Improve hemodynamics in experimental pulmonary arterial hypertension; Ameliorate the impaired alveolarization and pulmonary artery remodeling	Jerkic et al., 2019; Park et al., 2019; Porzionato et al., 2021
	Acute Respiratory Distress Syndrome	Mitochondrial transfer via tunneling nanotubes to enhance macrophage phagocytosis.	Improve bacteria clearance rate, reduce disease response, and have obvious antibacterial effect	Jackson et al., 2016; Morrison et al., 2017; Huang et al., 2019
Wound healing	Diabetic wound healing	MSC polarizes macrophages to M2 type through MSC-Exos and PGE2- dependent pathways	Relieve inflammation and autoimmune response	Philipp et al., 2018; Saldana et al., 2019; Liu et al., 2020
	Cutaneous wound healing	Inhibit NF-kB pathway to promote the polarization of M2 macrophages	Promote cutaneous wound healing, reduce scar areas and the infiltration of inflammatory cells	Lee et al., 2015; Ti et al., 2015; Ko et al., 2020
Cancer	Suppress leukemia	Reprogram macrophages to the arginine-1 positive phenotype.	Change the bone marrow microenvironment and inhibit the development of leukemia.	Jafarzadeh et al., 2019; Nwabo Kamdje et al., 2020; Xia C. et al., 2020
	Breast cancer	Secrete exosomes to promote myeloid cells into M2-polarized breast cancer macrophages	Drive accelerated breast cancer progression	Wolfe et al., 2016; Francois et al., 2019; Wang et al., 2019
	Lung cancer	Increased miR-21-5p delivery by MSC-EV after hypoxia pre-challenge by reducing apoptosis and promoting macrophage M2 polarization.	Promote lung cancer development	Gazdic et al., 2017; Ren et al., 2019
Bone	Bone tissue repair	Secrete TGF-β to promote the transition from M1 to M2 macrophages; the transition of M1 to M2 is beneficial of proliferation and osteogenic differentiation of MSCs	Initiate bone regeneration and promote bone tissue repair	Gong et al., 2016; Zhang et al., 2017; Deng et al., 2020; Kang M. et al., 2020
*In vitro*	Mouse-derived bone marrow MSCs are cocultured with macrophages	MSC stimulates macrophage secretion of IL-10 through PGE2; TNF-α and iNOS expressed by macrophages are necessary for MSCs to secrete PGE2.	Reduced mortality and improved organ function and cultured banked human BMSCs may be effective in treating sepsis.	Nemeth et al., 2009
	MAPC cocultured with monocytes	Macrophage cytokines can produce inflammatory cytokines to stimulate MSC to produce PGE2 and blood cytokines IL-1RA	Suppress the IL-7-dependent T-cell expansion	Reading et al., 2015
	Coculture of MSC and macrophage in Murine inflammatory environment	Inducing macrophages to produce IL-10 is also partly involved in the beneficial effects of MSC-Exos	Reduce murine colonic inflammation	Liu H. et al., 2019
	MSC coculture with pro-inflammatory macrophage	Promotes the interaction of CD200 and CD200R on MSCs and the anti-inflammatory transformation of macrophages increases the production of TSG-6 by MSC	Enhance the inhibitory regulation of MSCs on T cells and macrophages	Li Y. et al., 2019

### Alleviating Autoimmune Diseases

Human gingiva-derived MSCs can enhance cutaneous wound healing by polarizing M2 macrophages (Vasandan et al., 2016; Zhao et al., 2016; Xu et al., 2017). In addition, melatonin (MT)-pretreated MSC-derived exosomes (MSC-Exos) can exert better effects on diabetic wound healing (Liu et al., 2020). MSC-Exos significantly inhibit macrophage expression of the proinflammatory factors IL-1β, TNF-α, and iNOS and simultaneously upregulate the anti-inflammatory factor IL-10, thereby increasing the polarization ratio of M1 to M2 to mediate the therapeutic effect (Philipp et al., 2018; Saldana et al., 2019). In colitis, MSCs are reported to regulate the polarization of M1 to M2 cells in a CCL2-dependent manner by upregulating the expression of IL-10 to alleviate intestinal inflammation (Giri et al., 2020). Liu H. et al. (2019) indicated that the systemic administration of exosomes from human bone marrow-derived mesenchymal stromal cells (BM-MSC-Exos) can effectively alleviate inflammatory bowel disease by interacting with colonic macrophages. Previous studies indicated that IL-1β derived from macrophages may stimulate MSCs to secrete IL-1RA and PGE2, which leads to the polarization of M2 macrophages, thereby forming a feedback pathway that enables MSC therapy improvement in hepatic injury (Bartosh et al., 2013; Lee et al., 2015; Reading et al., 2015; Luz-Crawford et al., 2016). Ko et al. (2020) reported that macrophage-derived amphiregulin is essential for MSC-mediated CD4+ T cell suppression and preserves epithelial stem cells to alleviate autoimmune uveoretinitis. More importantly, MSCs alleviate inflammatory injury depending on the presence of macrophages in the body, and pharmacological depletion of macrophages weakens the therapeutic effect of MSCs (Ko et al., 2016; Giri et al., 2020).

### Hematopoietic Microenvironment

Mesenchymal stem cells and macrophages are pivotal for the repair and maintenance of the bone marrow microenvironment. Macrophages can be reprogrammed by MSCs to an arginine 1-positive phenotype and have tissue repair functions, and these functions are extremely important in the leukemia microenvironment. The reprogramming of macrophages by MSCs changes the bone marrow microenvironment and inhibits the development of leukemia (Xia C. et al., 2020). In the leukemia microenvironment, leukemia cell exosomes change the immune characteristics of MSCs and macrophages (Jafarzadeh et al., 2019; Nwabo Kamdje et al., 2020). In addition, MSCs can reprogram macrophages into M2 macrophages with phagocytosis functions and can reduce the secretion of proinflammatory factors to control the tumor microenvironmental immunity (Wolfe et al., 2016; Francois et al., 2019; Wang et al., 2019).

### Bone Tissue Repair

It is worth noting that MSCs and macrophages are also extremely important in bone repair, where macrophages regulate the osteogenic differentiation of adipose tissue MSCs and their transition from M1 to anti-inflammatory M2 is beneficial to the proliferation and osteogenic differentiation of MSCs (Zhang et al., 2017). Moreover, macrophages can promote the osteogenic differentiation of MSCs by secreting extracellular vesicles to promote bone regeneration (Kang M. et al., 2020). Gong et al. (2016) also found that macrophage polarization plays an extremely important role in regulating MSC osteogenic differentiation to maintain bone homeostasis and bone regeneration. In addition, the MSC-derived extracellular matrix alleviates macrophage inflammation to promote bone regeneration (Deng et al., 2020). Other studies have shown that for certain materials, the interaction between MSCs and macrophages includes the MSC-induced transformation macrophage phenotypes and macrophage-induced promotion of MSC differentiation; therefore, the physiological functions of MSCs and macrophages are regulated to improve osseointegration and promote bone repair (He et al., 2019; Mahon et al., 2020).

### Myocardial Function Repair

Dichloromethane (DCM) rats have abnormal lipid metabolism and cardiac inflammation. MSCs improve metabolic abnormalities and downregulate blood glucose levels by increasing M2-phenotype macrophages to alleviate cardiac inflammation in DCM rats (Sun et al., 2018; Jin et al., 2019). Yan Liao’s laboratory also reported that MSCs have a promising therapeutic effect on myocardial infarction by promoting the polarization of M2 macrophages by secreting periostin (Liao et al., 2020).

In summary, in various diseases, MSCs interact with macrophages to adjust the inflammatory microenvironment and thus affect the occurrence and development of the disease.

## Conclusion

Mesenchymal stem cells are an emerging treatment for inflammatory and degenerative diseases, and they are entering early phase clinical trials. MSCs can regulate the biological function of innate and adapted immune cells. Macrophages are important innate immune cells that are found in nearly all inflammatory tissues, where they play a key role in maintaining normal tissue homeostasis. Evidence was provided showing that MSCs regulate the chemotaxis and function of macrophages and MSC-derived signals can contribute to disease remission by modulating macrophage function in certain cases (Figure 2). However, the exact role of MSCs in regulating macrophages remains to be determined.

The effects of MSCs on macrophages include inducing the transition from the proinflammatory M1 phenotype to the anti-inflammatory M2 phenotype, which reduces the secretion of proinflammatory factors and increases the secretion of anti-inflammatory factors. Moreover, MSCs could reduce the recruitment of proinflammatory macrophages to the site of inflammation. Furthermore, MSCs reciprocally regulate macrophage function. Studies investigating how macrophages influence the function of MSCs have gradually increased. Macrophages have a feedback effect on MSCs that includes differentiation, migration, apoptosis, and immunomodulatory functions. The synergistic effect of macrophages and MSCs maintains microenvironmental homeostasis.

At present, certain problems remain in the research on MSC and macrophage interactions. Soluble factors secreted by MSCs have been proven to have different effects on macrophages. The biological functions mediated by MSCs on macrophages vary with different inflammatory conditions. Further studies are needed to discover reliable markers for defining different subpopulations of macrophages, clarify the heterogeneity among different subpopulations of macrophages used for specific treatments and clarify the potential mechanisms by which MSCs regulate macrophages. Based on the current literature, the following two strategies to enhance MSC efficacy by targeting macrophage-MSC crosstalk can be outlined: (1) modulating the number of macrophages at the inflammatory site and (2) modulating the injection methods and dosages of MSCs in inflammatory diseases.

Research on the immunosuppressive effects of MSCs is underway, but additional research is required to clarify the interaction between MSCs and macrophages and identify the mechanism by which MSCs regulate macrophages to provide better solutions for the treatment of disease. In clinical applications that combine MSCs with M2 macrophages for the treatment of immune diseases, the stability and flexibility of the treatments should be closely considered and optimized to achieve the appropriate modulation of inflammatory responses at different stages of disease progression.

## Author Contributions

DL and YX searched the literature and wrote the manuscript. QL and QZ critically revised the manuscript and final approval of the work. All authors contributed to the article and approved the submitted version.

## Conflict of Interest

The authors declare that the research was conducted in the absence of any commercial or financial relationships that could be construed as a potential conflict of interest.
